# Medical Reform

**Published:** 1840-01

**Authors:** 


### PART FOURTH.

### Medical Intelligence.

---

### MEDICAL REFORM.

For upwards of thirty years has the attention of the medical profession been directed, with little intermission, to the necessity of obtaining from the legislature some reform of its condition regarded as a branch of civil polity About the year 1807, Dr. Harrison, then of Horncastle, in Lincolnshire, excited very general attention to the subject of medical reform ; and from that period to the present it has been the theme of discussion. This must suffice to show that the propositions for reform made at the present day cannot justly be regarded by any as taking the profession by surprise. If there be any who can now feel surprise at this subject being agitated, the regard of such persons for the general welfare of the profession must be slight, else they could not be ignorant, either of the manifold evils which beset it, or of the reiterated endeavours made to unite its members in an appeal to the legislature for the reforms needed. For the diversity of opinion which prevails on the necessity that exists for any reform, a brief explanation may suffice. They who thirty years ago advocated reform are either dead or superannuated, or, through long despondency of success, are become indifferent to what ceases to be to them a subject of any personal interest. A new generation has sprung up to whom the subject is less familiar, and many of whom have acquired predilections and preferences for certain corporate institutions by which their judgments are biased, and which disincline them to any change by which their favourite colleges would be lowered in estimation or influence, or their own private interests affected. Many of the latter, it is to be feared, will, on the present occasion, coalesce with the anti-reformists, or so coldly espouse their own cause as rather to clog the wheels of reform than to expedite their movements. In order that all may have the opportunity of viewing the subject in its several bearings, we purpose presenting to our readers a succinct but, we trust, accurate sketch of what requires to be known in order to form any rational judgment on the subject of medical reform, namely, the nature and extent of the evils which exist; the endeavours hitherto made to correct those evils; and the proposed measures by which it is contemplated to effect their extinction. With this summary, which, we trust, will put all concerned in possession of, at least, the elements of this question, we purpose dismissing it, for a time, from our pages;—leaving the regeneration of the profession to that body whom it most concerns, and who, if inclined to support their own interests, must be fully competent so to do—namely, the collective profession.

In order to have a clear conception of the present condition of the profession, it is necessary to look back for some centuries to the time when anything like a legalized medical profession was first constituted in these kingdoms. And this retrospect is the more necessary from the extraordinary fact that, by laws passed in the fifteenth century, does one most important branch of the profession continue to be governed at the present day! To the establishment of the London College of Physicians by the charter of Henry VIII., and the confirmation of this by subsequent statutes, may we assign the first commencement of a legalized medical profession in these kingdoms. At this period physic was paramount, and surgery only a subordinate branch surrendered to the fostering care of the barbers. Yet was not the latter wholly disregarded, for the charter of Henry gives to the college a power of licensing in surgery as well as in physic. On the policy pursued by this body in administering its chartered powers, it would be needless and irksome here to dwell. Suffice it to say that, instead of combining and consolidating even that branch of the profession which it was specially appointed to superintend, it limited the powers and privileges of the corporation to a few, placing the great mass of physicians who consented to acknowledge its authority in the subordinate and degraded class of Licentiates, endued with no corporate rights, and deriving from their connexion with the college only leave to practise their art, which the license of the college graciously permitted them to do, "saltem in nonnullis curationibus." A large portion of regularly-educated physicians have at all times dispensed with its license, and, upheld by the public, have practised without its authority, although in so doing they have acted illegally, and rendered themselves liable to pains and penalties on the information of any *qui tam* informer. So far as this branch of the profession is concerned, it assuredly needs some reform.

Surgery, as a distinct branch, was much later in receiving civic recognition and corporate rights than physic, for not until 1745, were the surgeons separated from the barbers, by 18 Geo. II. c. 15; and their incorporation as a Royal College did not take place until 1800. Much has been done by this body to improve the profession of surgery, but its chief efficiency has resulted from the moral influence which it possesses, beyond which it has no power, having no authority to interfere with the surgical practice of any one who, however unqualified, may choose to practise the art. Such defect alone furnishes decisive evidence of some reform being necessary in this body also.

Both these corporate bodies, in attending to what they deemed the duties and interests of their respective faculties, overlooked a class of the profession more numerous than both the others united, so essential to the public as to be of rapid increase; and, though not recognized by any chartered company, yet firmly established in the free exercise of their calling; the peculiarity of this class being that they dispensed medicines as well as prescribed. This class, now well known under the denomination of General Practitioners, was composed of all varieties; of surgeons who were compelled, in self support, to combine pharmacy with their more immediate art; of apothecaries, equally obliged to prescribe as well as dispense; even physicians have found it expedient to hold the doctorate in abeyance, and engage in the more certain and more profitable occupation of general practice. But this class being open to all who choose to enter it, no qualification being legally enjoined, its unprotected state excited general interest, and a legal constitution was given to it by the statute of 1815, called the Apothecaries' Act. The history of this measure claims grave consideration. The parties who originally sought it were anxious to procure the sanction and co-operation of the established medical institutions, and, accordingly, they applied to the Colleges both of Physicians and of Surgeons, to aid their endeavours; but both declined, the views which then prevailed causing both these bodies to disdain any connexion whatever with pharmacy. The preposterousness of this objection on the part of the surgeons was sufficiently egregious, for their own body had long supplied a large proportion of the general practitioners of the kingdom. In yielding to a false pride on this occasion, too, they inflicted a signal injury on the whole body of their own licentiates; for these, who, previously to the passing of the Apothecaries' Act, in 1815, had full power to practise pharmacy, were, after its enactment, restrained by it from all practice of pharmacy unless they possessed the license of the Apothecaries' Company, which license could only be obtained through apprenticeship, a given course of study, and formal examinations. It is clear that if the College of Surgeons had deemed the subject worthy of their notice, they must have had sufficient influence to procure the insertion of a clause into the Apothecaries' Act exempting their licentiates from its operation. Having neglected so to do, the consequence has been that all who have become general practitioners since 1815 have been obliged either to undergo a double examination, in order to obtain the license of the Apothecaries' Company as well as the diploma of the College of Surgeons, (legal necessity always requiring the former, and regard to public opinion frequently the latter,) or to content themselves with the Apothecaries' license only, the obtaining of which involves no examination in surgery, and the possession of which, consequently, implies no necessary knowledge of this branch of the science! The proportion of general practitioners who have entered into practice since 1815, who are licentiates of the Apothecaries' Company only, is very considerable, as every one knows. Such being the present state of this department of the profession, it must be allowed that it, too, stands in need of some reform.

But if each department of the profession thus prove to be defective, and in need of amendment, it is difficult to conceive the grounds on which any member of the profession could venture to assert that no reform is required. Every member must belong to some one of the departments mentioned; in his own department, at least, he must be sensible that all is not perfect; and unless his judgment be that acknowledged imperfection ought not to be rectified, he must unequivocally admit that some amelioration ought to take place. The hesitation occasionally evinced to acknowledge that reform is at all needed, proceeds, we suspect, not from any want of conviction of some reform being required, but from disinclination to acknowledge any plan of reform as suitable, save that which would aggrandise the special department to which the individual belongs. Each would readily allow reform to be expedient if the reform were confined, or even more especially directed, to the evils which beset his own department; but finding more comprehensive views prevail, and the interests of other departments advocated as claiming just support, he shrinks from bringing into hazard the rights and privileges which his own department possesses, and, unable to foresee all the consequences of change, he decries all reform. Many ingenuous minds yield to this feeling, and are perfectly honest in so doing. They, by a very natural partiality, are accustomed to deem their own department the most important of the whole; so regarding it, they as naturally consider that any damage sustained by it must be an injury to the collective profession, nay, to the whole community; and, from their best feelings, and with conscious integrity, they denounce all measures for a general reform as rash and hazardous. We trust that by showing each department of the profession separately to be imperfect, and in need of amendment, we have sufficiently established the main principle on which all reforming projects require to be founded, namely, that some reform of the profession is needed.

Our next enquiry might be into what each department has yet done towards perfecting itself even within its own limited sphere, to which alone have any endeavours been hitherto directed, no attempt having ever been made to bring the several branches into union and harmony with each other. But it would be a profitless task,—for we trust, ere we close this brief article, to show that no amendment of separate institutions which zeal, judgment, energy, and sincerity united could effect, would answer the end which the well-being of the collective profession, and the interests of the community imperatively demand. The old arrangement of the profession, derived from influences foreign to either its natural tendencies or its beneficial applications, comprised physicians to prescribe for internal diseases,—surgeons to deal with maladies calling for manual dexterity,—and apothecaries to dispense the prescriptions of both. Analogously to what may be observed in various other social constructions, the form of the edifice remained long after the appropriation of the several constituent tenements had varied immeasurably from their original destination. At present, we suppose, it is hardly within the memory of any one to recall the time when the foregoing adjustment of professional duties was strictly adhered to. Surgeons have long found their most considerable, and most profitable occupation to consist of the practice of physic rather than of surgery; and apothecaries have as successfully combined the practice of both physic and surgery with their more immediate art. Is this a ground of reproach? Far from it; both surgeons and apothecaries, in yielding to a public necessity which they could not, if they would, control, have but discharged their highest duty, that of ministering to the public welfare; and their triumphant encroachments on the department of the physician only prove how powerless are legal provisions and collegiate restrictions, when not based on the only sure foundations of sound principle and the public good. Sound principles give no sanction to the older division of labour in the medical profession, at least none to the extent to which it was proposed to be carried; nor was the public benefited by it. The public could not recognize any sufficient grounds for the distinctions made between physic and surgery, the human body, with all its properties and attributes, physiological and pathological, being the subject on which both were to be exercised. They could see no propriety in having a surgeon to take charge of a fractured limb, and a physician to minister to the fever which the accident occasioned; and they acted as common sense directed,—for, as the physician was not prepared to treat the fracture, they obliged the surgeon to grapple with the fever. Nay, they have done more; for, not content with requiring the surgeon to act as physician, they have, in a wide range of practice, forced him to become apothecary also, so far as to dispense the necessary medicines to his own patients. They who charge surgeons and apothecaries with having culpably and fraudulently trenched on the physicians' province are guilty of a great mistake, and of actual injustice. By no wilfulness, or fraudulent encroachment, could the change have been effected, if the public will and the public necessities had not enforced and rendered it inevitable. Speculations, however, on how the general practitioner of the present day has been called into existence, are out of date; the fact being, not only that this class actually exists to a wide extent, but that it is now legally recognized, and protected by legislative enactment far more perfectly than any other department of the medical profession.

Such being the indisputable facts, it is strange that any can be found to regard a renewal of the old system practicable, or even desirable. Yet are there several, chiefly among the class of physicians, who, yearning for a return of the good old times, really imagine that the old system could be revived, and whose beau ideal of medical reform would be the re-establishment of the physician, surgeon, and apothecary (*i. e.* druggist), each restricted to his own special functions, as was presumed to be the case in the earlier period. Far different is the reform of the profession now indicated. No reform, indeed, can have a chance of permanency, unless, while it provides for the welfare, efficiency, and respectability of the profession, it also adapts itself to those wants of the public which have been so unequivocally demonstrated: namely, by supplying an adequately qualified class of general practitioners.

Before discussing the special measures of reform which now seem most expedient, it is necessary to advert to one more circumstance, which, above all others, renders a legislative reorganization of the profession a matter of indispensable necessity, namely, the various and incongruous modes by which medical practitioners now procure admission into the profession. So numerous are these, that testimonials of qualification are now attainable from sixteen different sources, if not more. Where the object is the simple one of providing for the public a practitioner qualified to treat the several derangements of health to which the human body is subject, such a diversity in the primary qualification is not only unnecessary but absurd. But, unfortunately, it is mischievous also; for, of each qualifying body, the interests depend on the number of candidates qualified: and, though on speculative grounds it might seem that, in the competition so excited, superiority of system and discipline would be sure to attract towards it the greater number of candidates for medical honours, decisive experience has proved that the facility of obtaining these honours has far greater weight in determining the choice; and that, in the main, that institution which grants its testimonials on the easiest terms will be sure to supply the greatest number of practitioners. On the effect of such competition in lowering the tone of the profession by deteriorating the quality of the members composing it, it is needless to descant. Such rivalship, although it may commence with high-toned feelings, and the laudable ambition to merit a preference by the superiority of the advantages afforded, yields almost of necessity to the force of more sordid considerations, and ends in bartering its wares for pecuniary gain, instead of honorable and elevating fame. This is no imaginary surmise, but a real fact; it having been explicitly acknowledged at the great medical congress held in Dublin in May, 1839, that the Dublin College of Surgeons, which had long prided itself on the superiority of its discipline, and the strictness of its examinations, had been actually compelled to abate in both, in order to prevent the Dublin schools being deserted for others where testimonials were of easier attainment. Should a system, not only leading to such evil, but actually enforcing it, be suffered to continue, it needs no prophetic inspiration to predict that the final issue must be the degradation of the profession, retrogression in professional competency and acquirement, and consequent injury to the commonweal.

By no means can this progress of evil be arrested, save by establishing a minimum of qualification, without which no member can be suffered to undertake the responsibilities of medical practice; and, in order to ensure this minimum being acquired, by ordaining that qualification for practice shall be conferred by one examining and licensing body only, in each division of the kingdom, uniformity of qualification and equality of rights and privileges being established throughout the whole. All entering the profession through the same course of instruction and examination, all would, of course, have their general competency adequately proved. In order to afford due encouragement for still higher cultivation, and for the exercise of superior talents, there should be a higher grade, to which those ambitious of it might ascend. Such an arrangement would fulfil all that has ever been accomplished by the old system, while the higher class, generated by the proposed plan, would have the signal excellence not only of high literary and scientific attainments, but the still more valuable requisite of maturity of practical knowledge.

These were the considerations which led the committee of the Provincial Medical and Surgical Association to recommend, and the Association itself to adopt, the late petition presented on their behalf to both Houses of Parliament, in which they pray for the establishment of one examining and licensing body for each division of the kingdom. Without such consolidation, it was the clear conviction of the framers of this petition, that no reform of the profession through mere modification of the existing institutions could be of the slightest avail; and believing this to be the grand requisite which could furnish the only sure foundation of any system of effective reform, they limited, and wisely as we conceive, the prayer of their petition to this one point.

Such a measure would not alone supply every want of the profession, but it is essential as a groundwork for every other amendment, and, with it gained, the profession could afterwards, and, with little aid from Parliament, would be equal to work out its own regeneration. The examining and licensing bodies, having no direct interest in medical schools, would recognize those only which could furnish proof of their capacity; and the profession educated under such a system, could be at no loss afterwards in providing for efficient self-government. For this purpose it would be only required to create one general Medical Institution or College, or whatever might be its name, for each division of the kingdom. This might in the first instance be formed by unions of the present Colleges of Physicians and Surgeons; and as all legally qualified practitioners should be admissible as a matter of right, and enrolled on verifying their testimonials, no unjust or capricious exclusion could take place. The self-elective system, too, in the appointment to offices, which obtains in all our present Colleges, to their own detriment and the scandal of the profession, could be readily superseded by a more popular mode of election, in which the elective franchise might be limited to practitioners of three, five, seven, or ten years' standing, or in any other way that mature reflection should most approve. It may be imagined that by depriving the existing institutions of the right to confer the primary qualifications, they would be rendered inert and useless. But so far from this being the case, release from the unsuitable office of examining and licensing is absolutely necessary for allowing them to attend to manifold duties which ought to engage their attention. It is apprehended, we know, that by withdrawing from existing institutions the right of examining and licensing, these institutions would, in the loss of their present fees, become ruinously crippled in their finances. But all such fear must be groundless ; for if a general medical institution or college were to discharge the many important duties which would of necessity fall to its share, adequate support must be furnished to it from some source or other, as certain, at least, as that from which the present incomes of the several colleges are derived. As all medical practitioners would necessarily become enrolled in such colleges, a moderate introductory fee would raise a large fund in the first instance; successive enrolments would add to this; and should deficiency of means be, nevertheless, experienced, the services rendered to the state would amply warrant a claim on the public revenue, which no statesman would reject.

There is one more view of the subject yet to take, which may possibly reconcile one class of objectors to the kind of reform now contemplated. The old system of physician, surgeon, and apothecary would in fact be revived, having only superadded to it the all-important class of general practitioners. Many physicians and surgeons of course would not engage in pharmacy: therefore apothecaries would still be needed ; and these apothecaries are, in fact, already numerous, being the chemists and druggists of the present day, who are precisely what the apothecaries originally were in days of yore. Why were the apothecaries withdrawn from their proper functions, and elevated into medical practitioners ? Simply because the want of the public for a general practitioner, though clearly manifested, was not supplied by those institutions which ought to have discerned the want, and provided for it. Having overlooked the want, having even doggedly refused to acknowledge it when pointed out to them with express solicitation for their aid in supplying it, the apothecaries had no alternative but to fill the chasm thus wilfully left void,—and from 1815 to the present day, they have done so in a manner which reflects on the Apothecaries' Company the highest credit.

But ought it to be inferred from this that the present Apothecaries' Company is the body from which the general practitioners of the kingdom ought to emanate? We hesitate not to answer most determinedly in the negative. The Apothecaries' Company ought not to have the powers which they now possess; and the legislature will greatly fail in its duty, if the qualification of the general practitioner be not transferred to a less exceptionable source. In this transfer, no injustice will be done to the Apothecaries' Company. Antecedently to 1815, the associated Apothecaries had no province, save some superintendence over the drug trade. Accident, or rather the apathy and supineness of the Medical and Surgical Colleges, required them at that time to undertake a higher function,—and meritoriously have they discharged it. But the legislature which assigned to them this novel office is not only at full liberty to transfer it to other hands, but bound by every sense of public duty so to do. In resuming their old functions this company would find ample occupation, for greatly does the drug trade of the kingdom need enlightened superintendence; and in correcting its errors and abuses the Company of Apothecaries would find enough to do. By the members of this company—new modelled, perhaps, and with new powers, and under a new name, as a College of Pharmacy,—should every dispensing chemist and druggist be examined and licensed; and to them, as the most competent judges, should all inspection of the drugs kept by dispensers be assigned. For such duties their capabilities would be acknowledged by all; and the faithful discharge of these duties would be a national blessing. Should any one, endued with peculiar foresight, surmise that a change similar to what took place among the older apothecaries, namely, that of conversion into medical and surgical practitioners, would still go forward among the chemists and druggists, our reply is, that such would be the difference of circumstances that no such tendency on the part of the latter need be apprehended. The apothecaries became general practitioners, because the pressure of public necessity forced them into an unoccupied void. An adequate class of general practitioners being provided, there would be no void into which the chemists and druggists could pass. Prescribe they would, and ever will, behind their counters; nor could any legislation, however stringent, or penal, prevent this. But they would not, to any extent, engage in actual practice: the competition of a numerous, well-qualified, and active body of general practitioners leaving them no prospect of the slightest success in any such attempt.

We have thus presented to our readers briefly, and, so far as our own knowledge extends, faithfully, the elements of a question which, regarded in its bearings on the well-being of the community, is second in importance to none. Earnestly do we recommend to all classes of the profession to give to it that dispassionate and mature consideration which the magnitude of the interests involved demands.

---

